# Neural and Response Correlations to Complex Natural Sounds in the Auditory Midbrain

**DOI:** 10.3389/fncir.2016.00089

**Published:** 2016-11-10

**Authors:** Dominika Lyzwa, Florentin Wörgötter

**Affiliations:** ^1^Department of Nonlinear Dynamics, Max Planck Institute for Dynamics and Self-OrganizationGöttingen, Germany; ^2^Physics Department, Institute for Nonlinear Dynamics, Georg-August-UniversityGöttingen, Germany; ^3^Bernstein Focus NeurotechnologyGöttingen, Germany; ^4^Institute for Physics-Biophysics, Georg-August UniversityGöttingen, Germany

**Keywords:** response correlations, correlated trial-variability, natural complex sound, inferior colliculus, vocalizations, multi-unit cluster, guinea pig

## Abstract

How natural communication sounds are spatially represented across the inferior colliculus, the main center of convergence for auditory information in the midbrain, is not known. The neural representation of the acoustic stimuli results from the interplay of locally differing input and the organization of spectral and temporal neural preferences that change gradually across the nucleus. This raises the question of how similar the neural representation of the communication sounds is across these gradients of neural preferences, and whether it also changes gradually. Analyzed neural recordings were multi-unit cluster spike trains from guinea pigs presented with a spectrotemporally rich set of eleven species-specific communication sounds. Using cross-correlation, we analyzed the response similarity of spiking activity across a broad frequency range for neurons of similar and different frequency tuning. Furthermore, we separated the contribution of the stimulus to the correlations to investigate whether similarity is only attributable to the stimulus, or, whether interactions exist between the multi-unit clusters that lead to neural correlations and whether these follow the same representation as the response correlations. We found that similarity of responses is dependent on the neurons' spatial distance for similarly and differently frequency-tuned neurons, and that similarity decreases gradually with spatial distance. Significant neural correlations exist, and contribute to the total response similarity. Our findings suggest that for multi-unit clusters in the mammalian inferior colliculus, the gradual response similarity with spatial distance to natural complex sounds is shaped by neural interactions and the gradual organization of neural preferences.

## 1. Introduction

**Figure 1:**
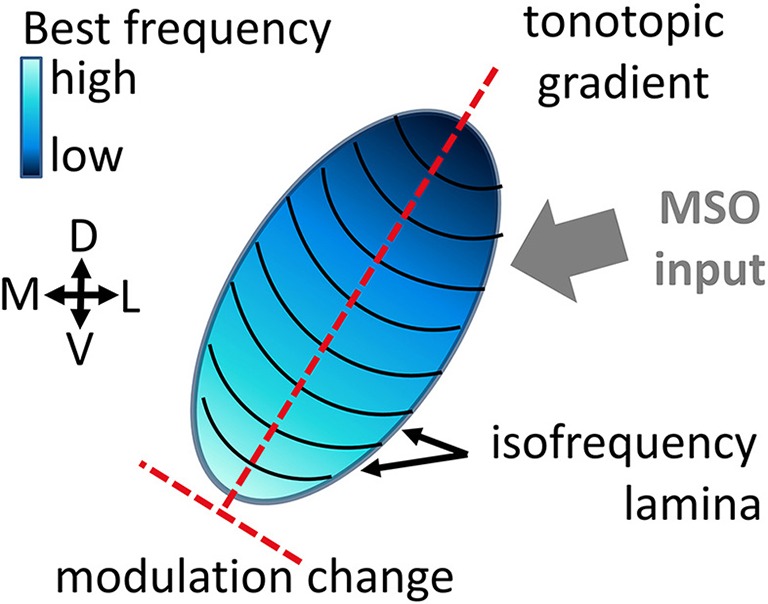
**Schematic of the central inferior colliculus displaying the tonotopic gradient and isofrequency laminae**. The inputs to the inferior colliculus are very diverse (Oliver, 2005), e.g., the Medial Superior Olive (MSO) projects mainly to low and middle frequency regions.

A neuron's response is shaped by all the inputs it receives, as well as by the integration and processing of these inputs, hence by the neuron's stimulus preferences. The inferior colliculus is the main center of convergence in the auditory midbrain (Irvine, 1992). It receives and integrates diverse preprocessed inputs from essentially all ascending auditory brainstem nuclei (Aitkin and Phillips, 1984; Malmierca et al., 2002) that terminate on different locations within the central inferior colliculus (ICC) (Oliver, 2005). Differences exist for e.g., high and low frequency regions, as well as for caudal or rostral regions. Information about interaural time differences from the medial superior olive for example is mainly projected to low and middle frequency regions (Oliver, 2005), see Figure 1. Neural preferences to stimulus frequency and modulation are mainly organized gradually within the ICC (Merzenich and Reid, 1974; Schreiner and Langner, 1988; Langner et al., 2002). In the tonotopic gradient low frequencies are represented dorsolaterally and high frequencies ventromedially (Rose et al., 1963; Merzenich and Reid, 1974). Along this tonotopic gradient, the stimulus frequency which elicits the highest spiking response gradually increases. For a given intensity this is called the best frequency (BF) and for the overall lowest spike-eliciting intensity this is the characteristic frequency (CF). Oriented approximately orthogonal to this frequency gradient are laminae that contain neurons with very similar best frequencies within a range of 1/3 octave, the isofrequency laminae (Schreiner and Langner, 1997). Strong indications for a concentric gradient within laminae of preferred amplitude modulation frequencies for the sound envelope have been provided (Schreiner and Langner, 1988; Langner et al., 2002; Baumann et al., 2011). The ICC has also been shown to be essential for extracting time-varying spectrotemporal information (Escabí and Schreiner, 2002) and therefore might be important for processing of complex sounds.

The neural response, the representation of acoustic stimuli in the ICC results from the interplay of the locally differing and heterogeneous input and the spatially-gradual change of spectrotemporal neural preferences. Along these gradients the sound is filtered either for the same spectral content or for the same amplitude modulations. Thus, the question arises, how for complex sounds, such as speech or vocalizations, the neurons' output is organized across this main convergence center. Neural interactions of synaptically connected neurons could further contribute to the specific organization of the neural representation in this nucleus, hence the output of the neurons to vocalization stimuli. Comparing response correlations and neural correlations which result from synaptically connected neurons and neurons receiving common input allows the evaluation of whether the similarity of responses is shaped by the underlying neural structure, rather than by the stimulus input to the neurons.

In this work we investigate the representation of vocalizations in the ICC by analyzing the dependence of the similarity of the recorded neural responses on the relative spatial distance between the multi-unit clusters. The hypothesis is tested that despite the locally differing various inputs, the spectrotemporal gradients induce a gradually changing neural representation of these natural complex sounds. To this end, neural responses are compared by cross-correlation for differently and similarly frequency-tuned neurons, with respect to the spatial distance between the neurons. Simultaneous and non-simultaneous recordings were compared to obtain indications of whether the similarity of recorded neural responses is mainly induced by the stimulus or by interactions of synaptically connected neurons and common input which can induce neural correlations. Whether neural correlations for vocalizations exist in the mammalian inferior colliculus has not been investigated before. Neural correlations can be beneficial, detrimental or have no effect on the encoding of sensory stimuli, and might depend on the specific neuronal structure (Averbeck et al., 2006).

We analyzed simultaneous recordings from 32 sites in the ICC of guinea pigs in response to monaurally presented conspecific vocalizations. The set of eleven behaviorally relevant sounds (Berryman, 1976) displays a wide spectrum of acoustic properties, such as amplitude and frequency modulations, harmonics and temporal correlations. It was suggested that neurons are adapted to process natural sounds (Rieke et al., 1995). Therefore, these might trigger responses which are not elicited by artificial or simple acoustic stimuli.

Similarity between the recorded responses from multi-unit clusters is obtained by pairwise cross-correlation analysis of the spiking activity i.e., the processed output of the ICC neurons. The correlation of spiking responses is additionally compared to the correlation of long range activity, the local field potential (LFP). Multi-unit cluster activity is the combined activity mainly from neighboring single neurons. This integrated activity could allow one to investigate local population processing in the ICC. It has also been shown that multi-unit clusters respond more strongly to natural sounds than single neurons (Grace et al., 2003) and that the natural stimuli can be more accurately discriminated based on these responses than based on single neuron responses (Engineer et al., 2008).

In a previous study of the mammalian ICC using dynamic moving ripple sound, Chen et al. (2012) investigated the correlation of single neuron spike trains with respect to dependence on the neurons' spectrotemporal properties. This study found that the best frequency is the most correlated parameter and a microcircuitry was suggested. In the present study, however, similarity is investigated at the level of multi-unit clusters which likely display a different correlation structure. Here, natural communication sounds are used instead of artificial sounds and a different spatial range of up to 1600 μm is probed. Dependencies on spatial distance have not been found for the grass frog midbrain (Epping and Eggermont, 1987), but have been shown in the primary auditory cortex (Eggermont, 2006). Since the grass frog midbrain displays a weak tonotopic gradient, these findings do not transfer to the mammalian inferior colliculus with a clear tonotopic organization and substantial differences in the neural structure.

In summary, we find that neural correlations exist in the mammalian inferior colliculus and that the neural and response correlations for spiking and long range activity gradually decrease with spatial distance for similarly and differently frequency-tuned multi-unit clusters. This suggests that the gradual neural representation of vocalizations is shaped by interactions between the neurons and their spectral and temporal preferences.

## 2. Materials and methods

### 2.1. Electrophysiology

Neural activity was collected from the central nucleus of the inferior colliculus (ICC) of adult male and female Dunkin Hartley guinea pigs. Recordings were acquired from 11 guinea pigs (458–749 g) in 3 to 4 electrode insertion positions (taken altogether 36 positions), with activity recorded simultaneously from 32 sites. The electrophysiological recordings and experimental setup are described in detail elsewhere (Rode et al., 2013; Lyzwa et al., 2016).

**Figure 2:**
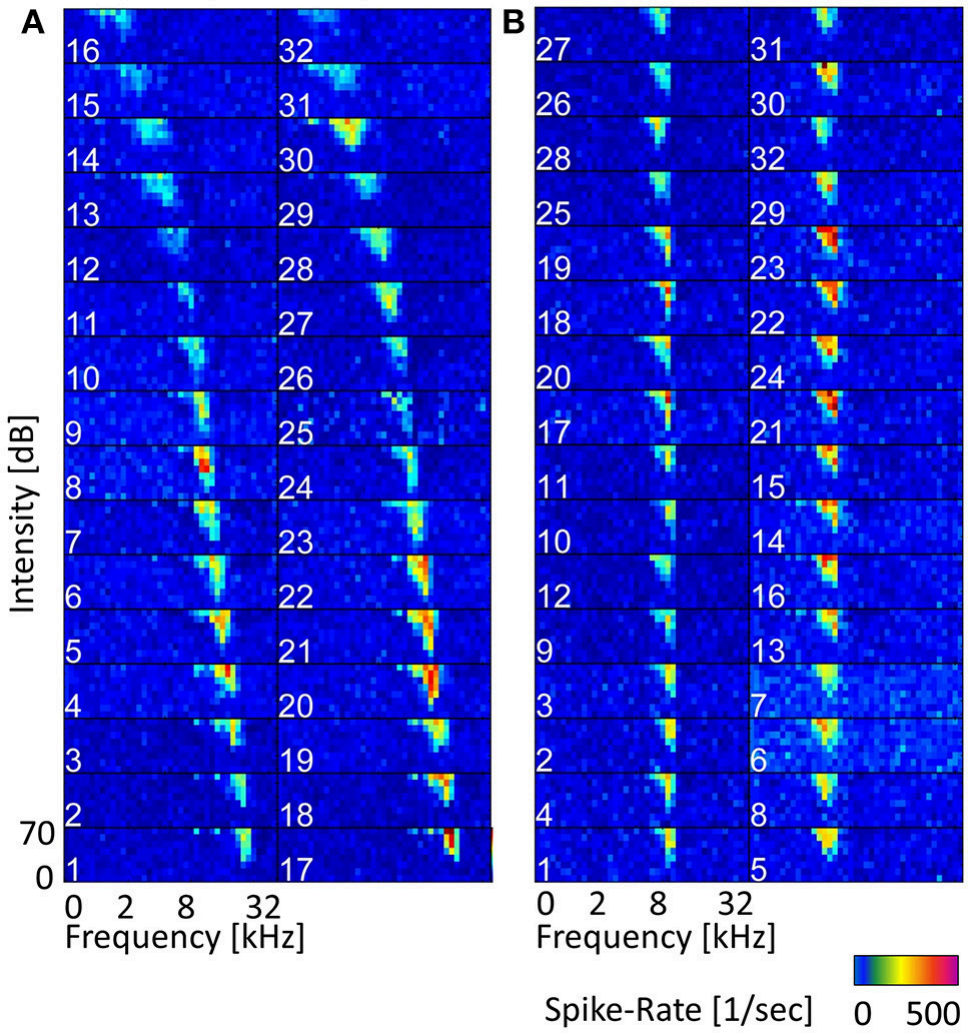
**Frequency tuning along the tonotopic gradient and within a few isofrequency lamina**. **(A)** Frequency response maps (FRMs) recorded from 32 sites along the tonotopic gradient with a linear double-shank electrode. The characteristic frequency (CF) covers a range from 0.5 to 29 kHz. The CF increases gradually from sites higher up (dorsolateral) to lower ones (ventromedial), also the shape of the FRM changes from a broader symmetric shape to an elongated and skewed shape for higher frequencies. The topmost sites do not show strong responses and might be lying outside the central IC. **(B)** FRMs of multi-units recorded with a double-tetrode electrode from two isofrequency laminae with CFs of about 7 kHz (left column) and about 4 kHz (right). The CF does not change visibly within one isofrequency lamina, but the frequency tuning varies in sensitivity, e.g., spike rates from site 23 and site 5 vary by over 150 Hz.

For the recording, either a linear double-shank array (shank distance was 500 μm with 16 contacts linearly spaced at 100 μm, on each shank) or a 4-double-tetrode array (shank distance of 500 μm, contact distance of 25–82 μm within a tetrode) were used to measure activity simultaneously from 32 sites (impedances were 0.5–1 MΩ at 1 kHz; NeuroNexus Technologies, Ann Arbor, MI). Whereas the linear-double shank array mainly records responses along the best frequency gradient, and covers a broad range of best frequencies, the 4-double-tetrode records from a few neighboring isofrequency laminae, and several multi-unit clusters that have similar frequency tuning (Figure 2). These units have similar best frequencies, but might have different preferences for amplitude modulations (AM) depending on their spatial distance within the ICC (Schreiner and Langner, 1988; Baumann et al., 2011). The electrode array was introduced under an angle of 45°dorsolaterally along the tonotopic gradient of the ICC, into 3 to 4 different insertion positions for each animal. The animals were anesthetized with an intramuscular injection of ketamine with 40 mg/kg and of xylazine with 10 mg/kg, and maintained in a non-reflexive state with periodic supplements (Rode et al., 2013). They were stereotactically fixed with ear tubes through which the sound was presented directly to the left eardrum. While acoustically presenting vocalization stimuli to the left ear, neural activity was recorded from the contralateral ICC at a sampling rate of 24.414 kHz using a TDT Tucker Davis System. For each vocalization 20 trials were recorded with intensities of 30–70 dB SPL in steps of 10 dB SPL. For the analysis of this study recordings are those at 70 dB SPL stimulus intensity, as these show the strongest response.

Frequency response maps (FRM) were obtained from spiking responses to pure tone stimuli. A total of 40 stimulus frequencies, ranging between 0.5 and 45 kHz, with a ramp rise and fall time of 5 ms each and a duration of 50 ms were presented. From the FRMs, the best and characteristic frequencies were obtained and ranged from 0.5 to 45 kHz. The frequency response maps for a linear double-shank recording along the tonotopic gradient and for a 4-double-tetrode recording are given in Figure 2.

### 2.2. Vocalization stimuli

**Figure 3:**
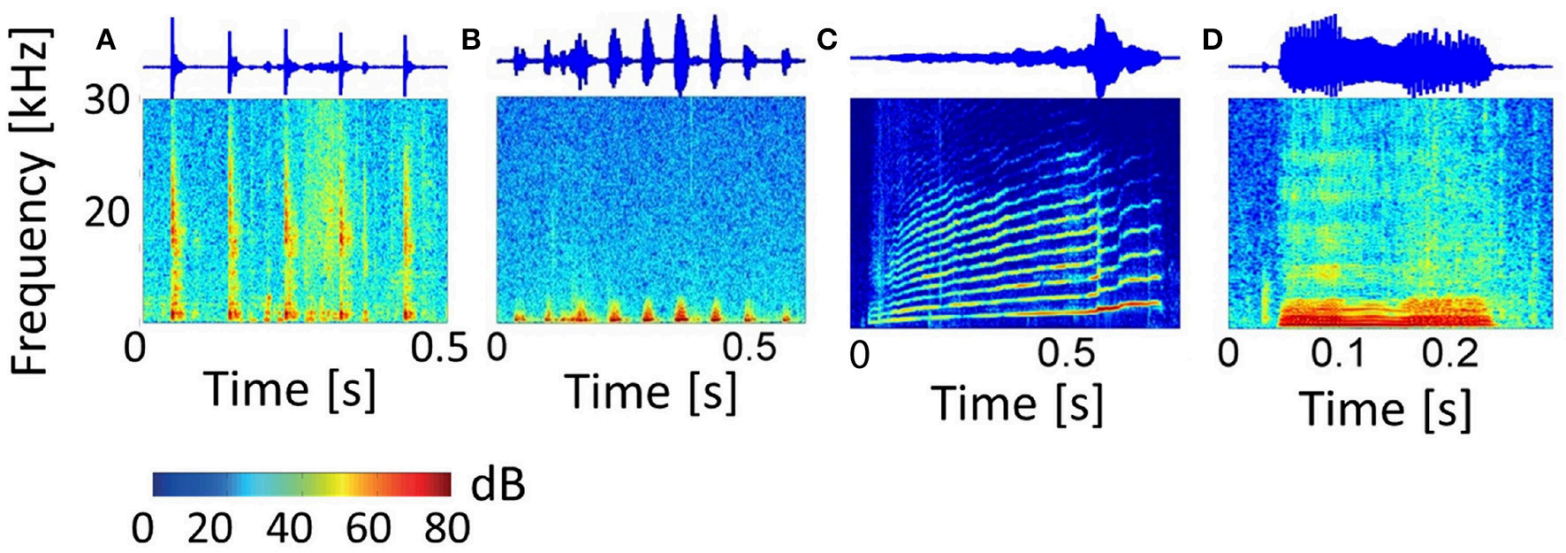
**Vocalizations**. Spectrograms and waveforms of four representative examples of the entire set of eleven guinea pig vocalizations. The “tooth chatter” **(A)** and “drr” **(B)** have periodicities in the waveform, with **(A)** containing frequencies up to 30 kHz, and **(B)** containing only low frequencies below 3 kHz. The “long scream” **(C)** and “squeal” **(D)** have complex waveforms, cover a broad spectral range and show harmonics.

The 11 vocalization stimuli used in this study are a representative set of guinea pig communication calls and give information about the animal's behavioral state (Berryman, 1976). The vocalization set includes the “long scream,” “short scream,” “squeal,” “tooth chatter,” “whistle,” “long chutter,” “short chutter,” “low whistle,” “low chutter,” “drr,” and “purr.” Figure 3 shows the spectrograms and waveforms for four examples of this set: the “tooth chatter,” “drr,” “long scream” and “squeal.” These natural complex sounds display a variety of frequency modulations, frequency ranges and envelope types. Some vocalizations such as the “drr” (b) contain mainly frequencies below 3 kHz and have periodicities in the waveform. The “tooth chatter” also has a periodic waveform but a frequency content of up to 30 kHz. Others, such as the “long scream” (c) and “squeal” (d), have complex waveforms, cover a broad spectral range and display harmonics. Vocalizations with a complex waveform and almost all energy at low frequencies are also present. The vocalizations were played 20 ms after recording onset and vary in duration between 300 ms and 1300 ms. They were recorded from male and female Dunkin Hartley guinea pigs at a sampling rate of 97.656 kHz. Details on the stimuli recording including sound calibration and the frequency content for the full vocalization set can be found in Rode et al. (2013) and Lyzwa et al. (2016).

### 2.3. Preprocessing of neurophysiological data

In order to investigate responses to vocalizations in the ICC, the spiking activity is employed for the analysis, because this is the processed output of the neurons (Pettersen et al., 2012). Additionally, the analysis is extended to the local field potential (LFP), which is long-range activity and contains i.a. the synaptic input to the neurons (Pettersen et al., 2012). The local field potentials were obtained by Butterworth filtering the voltage traces in a range between 0.5 and 500 Hz (Pettersen et al., 2012).

To obtain spiking multi-unit activity the recorded voltage traces are Butterworth-filtered with a passband of 300–3000 Hz and thresholded at *z* = 3 standard deviations exceeding the ongoing activity (Θ = μ + *zσ*), with the mean μ and the standard deviation σ of the ongoing activity. This spontaneous or ongoing activity was acquired from the first 20 ms of each recording, during which no stimulus was presented, in order to account for adaptation effects over time and different spontaneous rates of the neurons. Due to the low impedance of 0.5−1*M*Ω, detected spikes likely originate from different single neurons, and therefore no refractory period between spikes was assumed. The recorded activity is multi-unit cluster activity, which is the compound spiking response mainly of several neighboring single neurons recorded from one site. We used the offline spike-sorting program *WaveClus* (Quian Quiroga et al., 2004) to sort and separate spikes according to the spike waveform on a subset of the passband-filtered multi-unit cluster responses, with details in Lyzwa et al. (2016). Separation into single units was not possible because within sorted clusters a significant fraction of the spikes displayed inter-spike-intervals of less than 3 ms; and in some cases spikes could not be sorted into clusters. The responses investigated here are from neural groups comprising at least 3–5 single neurons and smaller contributions from neurons that are farther away from the recording electrode and are not distinguishable. Note that it is possible that different sub-groups of multi-unit clusters respond to different vocalizations.

Multi-unit spike trains were binned at 1 ms and convolved with an exponential filter function, *f*(*t*) = *t*·*exp*(α·*t*), with time *t*, to mimic the time course of excitatory postsynaptic potentials (EPSP) (van Rossum, 2001), as used by Machens et al. (2003). The full width at half maximum α, of the EPSP-like function was chosen to be 3 ms. The convolution was chosen with the respective window of 3 ms, because a response window of 3–10 ms has been shown earlier by Machens et al. (2003) to be better discriminated than for higher or lower resolutions for spike responses to communication sounds. The lower limit of the suggested interval had been chosen as it has the highest resolution. In an extensive previous study, several metrics to compare the similarity of neural responses for the present big multi-unit cluster set had been carried out, some are displayed in Lyzwa et al. (2016). For spiking activity, convolution with this filter function proved to be the most discriminative one across this data set. Averaging the binned spike trains across all *n*_*trial*_ = 20 trials yielded the post-stimulus time histogram (PSTH).

### 2.4. Cross-correlation analysis

In order to test the similarity of responses from different multi-unit clusters to the same vocalization, we employed cross-correlation which yields a compact description for the large set of neurons analyzed in this work. The similarity of responses to each vocalization was tested for pairs of multi-unit clusters from one recording which allows one to include the spatial distance, given by the electrode array, between the neurons in the analysis. This correlation-based similarity measure of spike trains (Schreiber et al., 2003) is one of several approaches. It had been employed earlier for neural discrimination of single neurons and groups of neurons (Wang et al., 2007) and has been shown to be a very discriminative measure for the present data set (Lyzwa et al., 2016). We computed the degree of correlation between the responses from different multi-unit clusters of one recording set to the same stimulus. Pairwise, EPSP-spike trains or LFPs from two multi-unit clusters *x*(*t*), *y*(*t*) of length *n*, were cross-correlated and the highest correlation value within a maximum possible delay of τ between the responses was selected:

(1) $$\text{Corr}(\tau )=\text{max}\left(\frac{\sum_{t\;=\;1}^{n-\tau }(x\left(t+\tau )-\langle x\rangle )\cdot (y(t)-\langle y\rangle \right)}{\sqrt{\sum_{t\;=\;1}^{n}(x\left(t\right)-\langle x\rangle {)}^{2}\sum_{t\;=\;1}^{n}{\left(y\left(t\right)-\langle y\rangle \right)}^{2}}}\right)$$

with a lag of τ = [−10 *ms*, 10 *ms*]. This delay is within the range of maximum response latencies in the ICC (Langner et al., 1987). The correlation values were computed with a lag, because response latencies do vary across multi-unit clusters with different spectral preferences (Langner et al., 1987). The correlation values of *n*_*trial*_ = 20 trials for one multi-unit cluster pair were then averaged. These response correlations (“*Corr*”) take into account the temporal structure of the responses and a delay between the two responses is possible, hence they are not “signal correlations” (Cohen and Kohn, 2011).

### 2.5. Neural correlations

Correlations between the recorded responses (spiking and LFP) of different multi-unit clusters from one set of simultaneously recorded sites are termed “response correlations” throughout this study. These response correlations (“*Corr*”), can contain stimulus and neural correlations.

Stimulus correlations can be present when a stimulus is applied and are due to the neurons responding to and following the same stimulus. Stimulus correlations should not be mistaken for correlations of the stimulus (or between different stimuli) but are increased correlations of the neural responses due to the same stimulus. These stimulus correlations are present for simultaneous and non-simultaneous recordings, as long as the same stimulus is used.

Simultaneously recorded spiking responses can show correlated trial-to-trial variability (Averbeck et al., 2006). This correlated variability which is termed neural or noise correlation, is due to synaptically connected, interacting neurons and neurons receiving common input. If no stimulus is present, the response correlations from the recorded neural activity are the neural correlations (*N*). Neural correlations can also be obtained from recordings for which a stimulus was applied. For stimulus-driven simultaneous responses, the measured response correlations contain, in addition to the neural correlations, also the stimulus correlations. In order to separate the stimulus correlations (*S*) from the response correlations (“*Corr*”), simultaneously recorded trials of the multi-unit clusters were randomly shuffled over trials with the same stimulus (Abeles, 1982) before correlating them, and thus only stimulus correlations remain. To obtain the neural correlation, the stimulus correlation is subtracted from the response correlation for each trial and the average is taken. This approach (Abeles, 1982) has been developed for single neurons, and attempts to infer functional connectivity from the computed neural correlations. Here, we use multi-unit clusters, and we do not attempt to make inferences about functional neural connections but to test whether neural correlations exist. If correlated neural activity is present then there is a significant difference between response correlations and the stimulus correlations. Significance was assessed using the Student's *t*-test for normal distributions, and the Wilcoxon-Mann-Whitney test for comparison of non-normal distributions.

In order to visualize the effect of neural correlations on the encoded stimulus information, scatter plots are often used (Averbeck et al., 2006). They display the distribution of averaged spike rates of two neurons or neural groups to different stimuli. The more the distributions of the responses to the different stimuli overlap, the less information is carried by them. Comparing the scatter plot of simultaneous trials to the one with shuffled trials yields information whether the neural correlation affect encoding. If, for example, separability of responses to different stimuli increases when removing neural correlations, these are detrimental for encoding.

### 2.6. Frequency tuning and spatial distance of neuronal pairs

Correlation is investigated for neuronal pairs of two multi-unit clusters. The multi-unit clusters are characterized by their spectral and spatial distance. The spectral distance is the difference measured in octaves between the characteristic frequencies of the two multi-unit clusters. Pairs differing by more than 1/3 octave in their characteristic are most probably from different isofrequency lamina along the tonotopic gradient (Schreiner and Langner, 1988). These pairs were assigned to the group of differently frequency-tuned neurons. Those pairs that have the same preferred frequency within an interval of 1/3 octave are likely from the same isofrequency lamina (Schreiner and Langner, 1988) and were assigned to the group of similarly frequency-tuned neurons.

The spatial distances between the multi-unit clusters of all pairs were mapped according to the channels on the electrode. Distances between all 32 channels were either obtained directly from the NeuroNexus manual (NeuroNexus Catalog, Ann Arbor, MI 48108, 2014) or calculated using the Pythagorean theorem, yielding a 32 × 32 matrix, respectively for the linear double-shank and the 4-double-tetrode electrode array. Minimum and maximum distances for the double-shank (D) and double-tetrode (T) array were respectively *D*_*min*_ = 100 μm, *D*_*max*_ = 1581 μm and *T*_*min*_ = 25 μm, *T*_*max*_ = 1372 μm. Only pairs with a minimum distance of 200 μm were considered for the correlation analysis of similarly tuned neurons (respectively 100 μm for differently tuned neurons) (Malmierca et al., 1995; Buzsáki, 2004), in order to assure that no multi-unit clusters are taken from adjacent recording sites, which possibly share the same neurons and therefore yield excessively high correlation values. Information on the multi-units' positions (e.g., histological stains) between different units is not available. For similarly frequency-tuned neurons, we excluded unit-pairs closer together than 200 μm; this length corresponds to the thickness of a laminae according to Malmierca et al. (1995), therefore, all pairs used for the analysis have at least some spatial separation orthogonal to the tonotopic gradient. Preferences for best amplitude modulation frequencies are organized concentrically within a lamina (Schreiner and Langner, 1988; Langner et al., 2002; Baumann et al., 2011). Hence, spatial distance for similarly frequency-tuned neurons likely implies a difference in best amplitude modulation frequency. The correlation analysis was performed for pairs from the same recording, yielding ideally (32 × 31)/2 = 496 pairs, which do not include autocorrelations or correlations counted twice. However, only pairs with a sufficiently large spatial distance of 200 μm (100 μm) were considered for the correlation analysis. The distribution of spatial distances was not uniform and also differed for double-shank and tetrode recordings. Therefore, the number of multi-unit clusters, for which correlation values were averaged for one spatial distance, varied across recordings. Differences in response similarity based on different amplitude modulation preferences for multi-unit clusters with the same frequency tuning might be averaged out when taking the mean across multi-unit pairs. Note that the use of multi-unit clusters for the study could limit the ability to assess the degree to which correlated firing may encode temporal features in the vocalizations.

Correlation is computed between multi-unit responses to the same vocalization, from one recording set which consists of responses from 32 multi-unit clusters. The analysis is repeated for all 36 recordings and analyzed for each recording. It was verified that the observed trend is consistent across recording sets. In the following, results for an individual example recording set are shown. Averages across all recording sets (1152 multi-unit clusters) were also taken. When averaging values across multi-unit pairs, the displayed error bars were chosen to represent one standard deviation to indicate correlation variability across multi-unit pairs. The error of the correlation values were computed via error propagation and are minor.

## 3. Results

We analyzed response, stimulus and neural correlations from 1152 multi-unit clusters across a wide frequency range of the central inferior colliculus of 11 guinea pigs for a spectrotemporally rich set of 11 species-specific vocalizations. Using cross-correlation, for spiking and LFP activity, we tested the variation of response similarity for individual vocalizations across the ICC and investigated whether correlation values depend on the spatial distance between multi-unit clusters. We compare response correlations and the contributions respectively due to the stimulus and due to neural interactions. At first, we display time-averaged neural responses to vocalizations, and then show correlations for similarly- and differently-tuned neurons.

### 3.1. Neural responses

**Figure 4:**
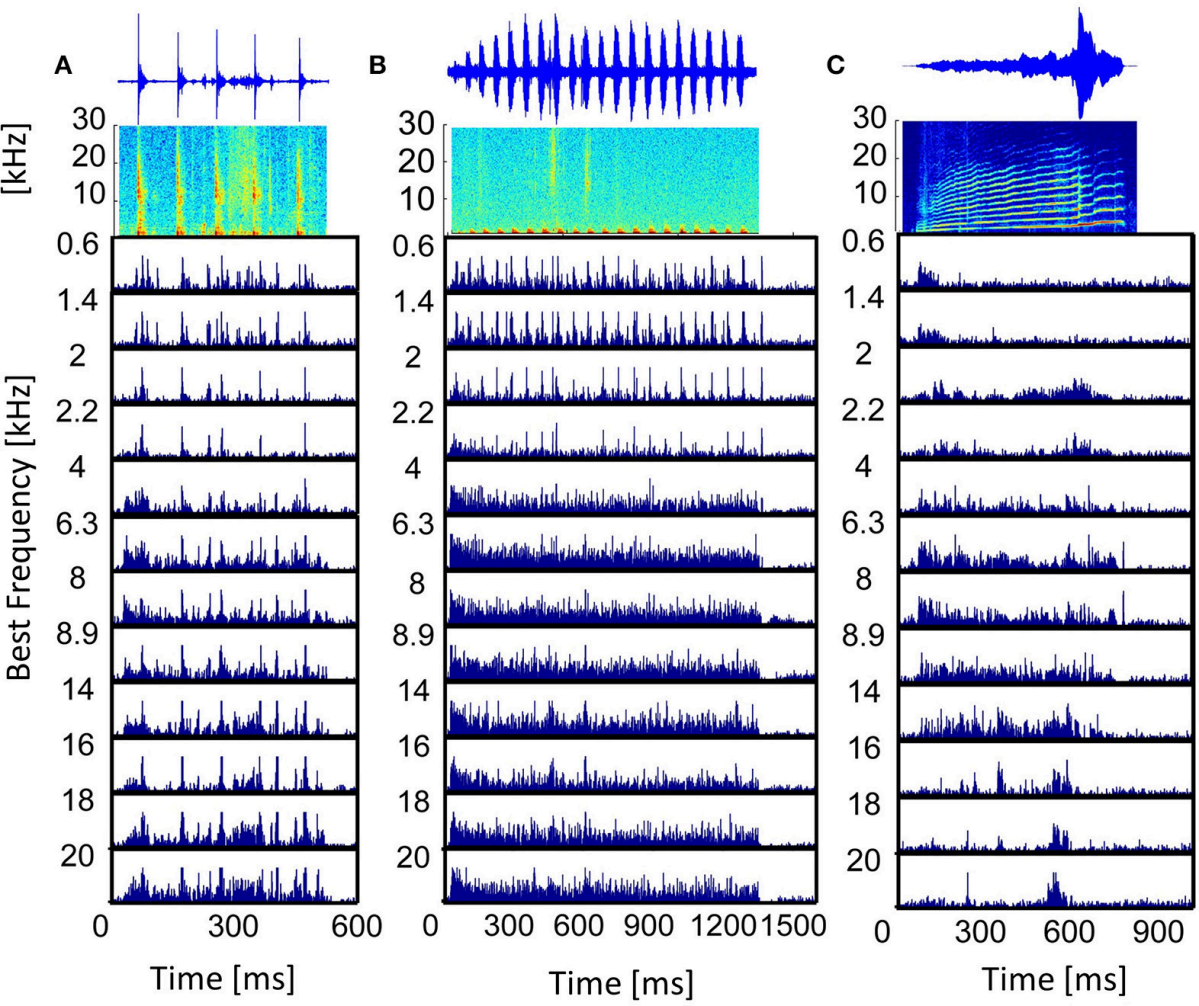
**Vocalization post-stimulus time histograms**. PSTHs in response to the vocalizations **(A)** “tooth chatter,” **(B)** “purr,” and **(C)** “long scream”; for multi-unit clusters from a linear double-shank recording, spanning a best frequency range of 0.6–20 kHz. On top, the waveforms and spectrograms are displayed.

The post-stimulus time histogram (PSTH) represents the trial-averaged (*n*_*trial*_ = 20) temporal neural response. Responses to vocalizations vary for differently frequency-tuned multi-unit clusters and follow the spectrally matching components in the stimulus. Figure 4 displays the PSTHs of multi-unit clusters along the best frequency (BF) gradient in response to three vocalizations. Responses to the “tooth chatter” phase-lock to the stimulus envelope throughout the whole best frequency range (Figure 4A), because the stimulus has spectral energy in this range (Figure 3A). However, the response in general becomes broader for high best frequency neurons. Responses to the “purr,” on the other hand, phase-lock accurately only for low frequency neurons, but then become broad and unspecific (Figure 4B). Spectral energy is present for frequencies up to 3 kHz in the “purr” vocalization. At the start and the end of the stimulus presentation, onset and rebound responses are more pronounced for middle and high best frequency neurons.

The responses' dependence on the match of the best frequency and the spectral content of the stimulus (Suta et al., 2003) is clearly illustrated by the PSTHs to the “long scream” (Figure 4C). In the beginning of the stimulus, only low frequencies are present, and only low-BF multi-unit clusters respond. Subsequently, the stimulus contains frequencies up to 25 kHz and middle-BF multi-unit clusters respond. High-BF multi-unit clusters respond to a high frequency peak at 600 ms. In some cases, these multi-unit responses can be approximated by the bandpass filtered waveform of the vocalization, filtered around the best frequency of the multi-unit cluster (Lyzwa, 2015), hence it seems likely that clusters with the same best frequency also have higher correlations of their responses. Similarity of responses to the same stimulus by different multi-unit clusters can be directly obtained by comparing the PSTHs. In order to quantify similarities across the large data set of multi-unit clusters used in this work, a more compact measure is employed. To this end, responses are cross-correlated and the correlation value indicates the degree of response similarity.

### 3.2. Dependence on similarity of frequency tuning

Response correlations to vocalizations are compared for multi-unit clusters with similar and different frequency tuning. In general, single and groups of neurons in the same isofrequency lamina have, within a 1/3 octave (Schreiner and Langner, 1988), the same preferred frequency but possibly different preferences for amplitude modulations (Schreiner and Langner, 1988; Langner et al., 2002; Baumann et al., 2011). Neurons with different frequency tuning properties are in different laminae of the tonotopic gradient.

**Figure 5:**
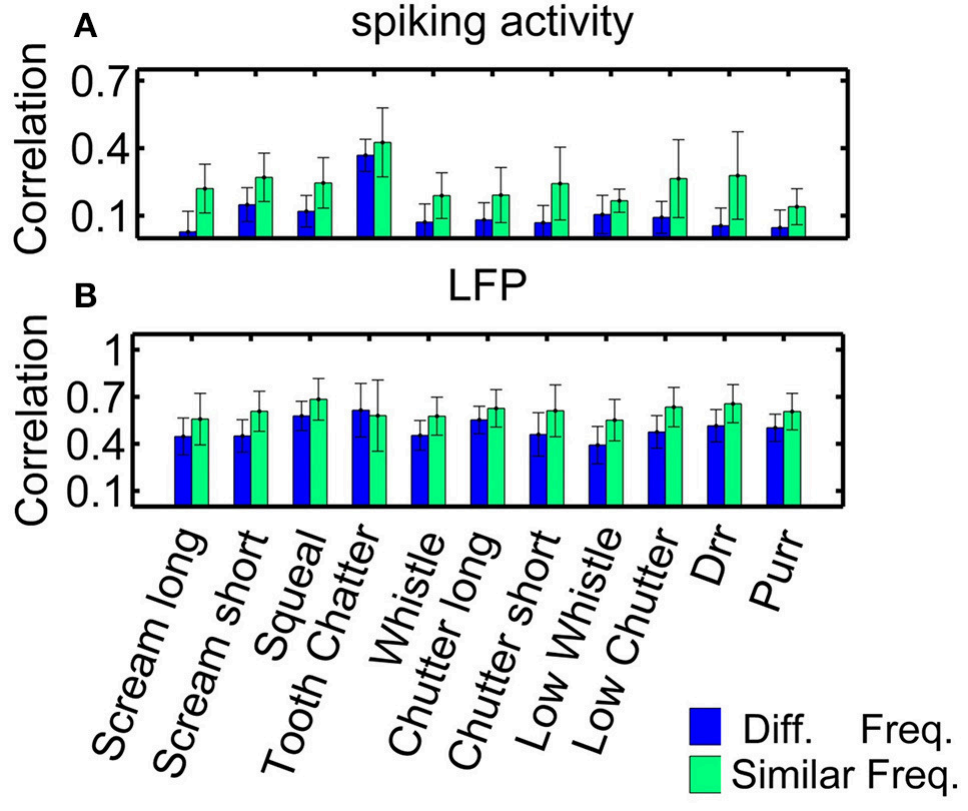
**Correlations for similarly and differently frequency-tuned neurons**. Averaged correlation values for multi-unit pairs from one recording (*n* = 155), for comparison, the number of pairs was kept constant for the two cases. **(A)** Spiking activity, **(B)** local field potentials. Responses for similarly frequency-tuned neurons are significantly more correlated than for differently frequency-tuned neurons (two-sided Wilcoxon-Mann-Whitney test *p* = 0.05).

As an example for multi-unit cluster pairs from one recording, Figure 5 displays the correlation values for each vocalization, for spiking activity and local field potentials. Correlation values show large variability within one recording set, as depicted by the error bars which correspond to one standard deviation. Correlation values are significantly higher for similarly frequency-tuned pairs than for multi-unit clusters with different best frequencies as assessed by the two-sided Wilcoxon-Mann-Whitney test, *p* < 0.05, for all vocalizations (except for the “tooth chatter” in LFPs). These correlation values vary across the different recording sets, because of the different distributions of frequency tuning similarity and spatial distances between the neuronal pairs.

Correlation values are significantly higher for LFP than for spiking activity for each vocalization, for similarly and differently frequency-tuned neurons (*p* < 0.05). The LFP responses are long-range activity, and spread throughout wider spatial and frequency regions than spiking responses which are confined to the range of one multi-unit cluster. The responses to the “tooth chatter” are significantly larger than to all other vocalizations, which is due to the stimulus' spectral content across a wide frequency range and the responses' phase-locking throughout this range (see Figure 4A).

Higher correlation within similar frequency regions than across the frequency gradient might point to vocalizations being processed by isofrequency laminae as functional units (Schreiner and Langner, 1997), however, even though differences exist, they are minor and variability exists across multi-unit clusters. The frequency selectivity made almost no contribution to the correlations between pairs of units. In the following, correlation dependence on spatial distance will be investigated separately for these two groups.

### 3.3. Dependence on spatial distance

**Figure 6:**
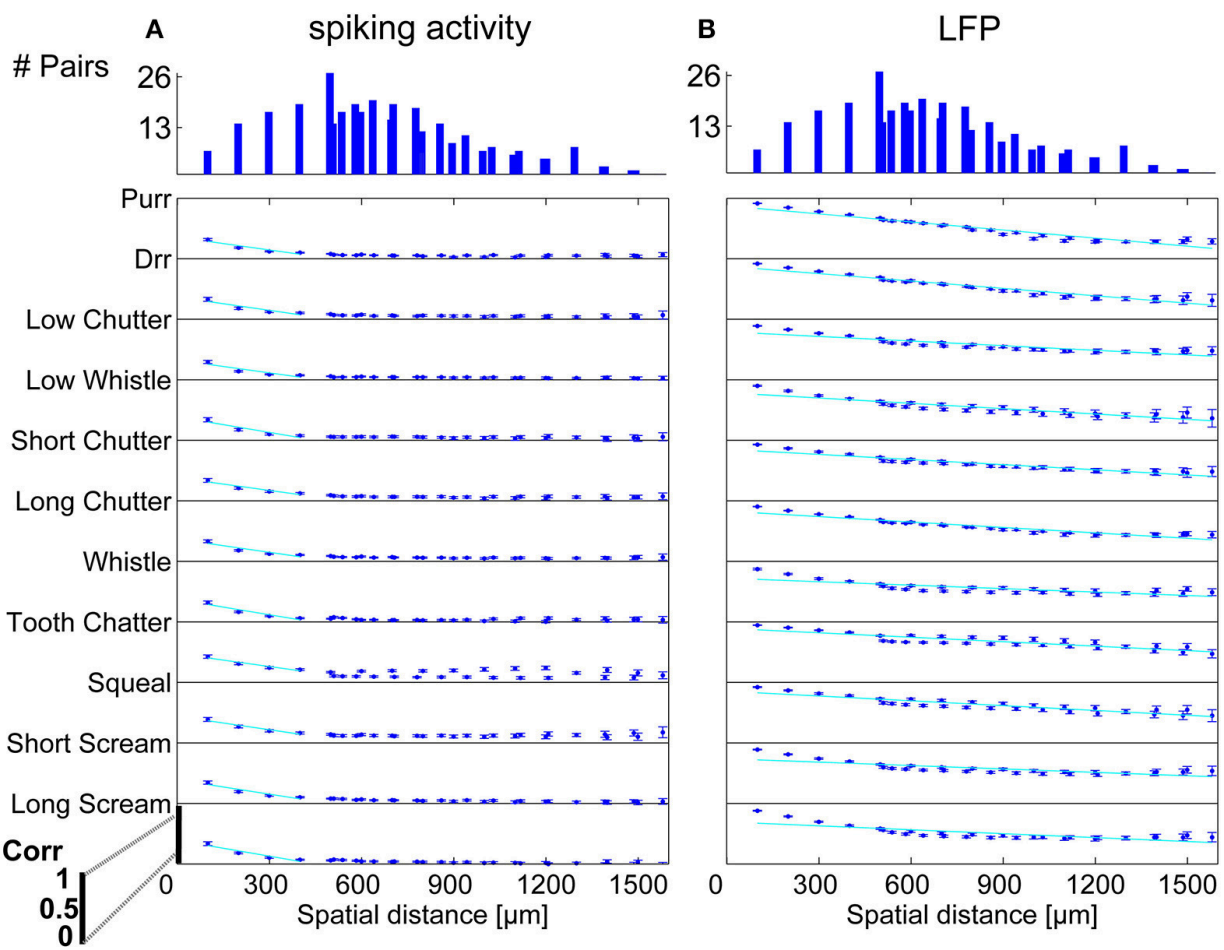
**Correlation dependence on spatial distance for differently frequency-tuned neurons**. Averaged correlations values for each distance from one recording set are displayed for all vocalizations, for **(A)** spiking activity, and **(B)** Local field potentials. Correlations decrease almost linearly with spatial distance, with a much bigger decrease for spiking activity than for LFPs. Exponential regression of this decrease (–), with overlaps of 61–100%, see Table 1. On top, the number of multi-unit pairs for each spatial distance over which the average correlation was computed is displayed. The y-axis for each vocalization shows a correlation range of 0–1.

**Figure 7:**
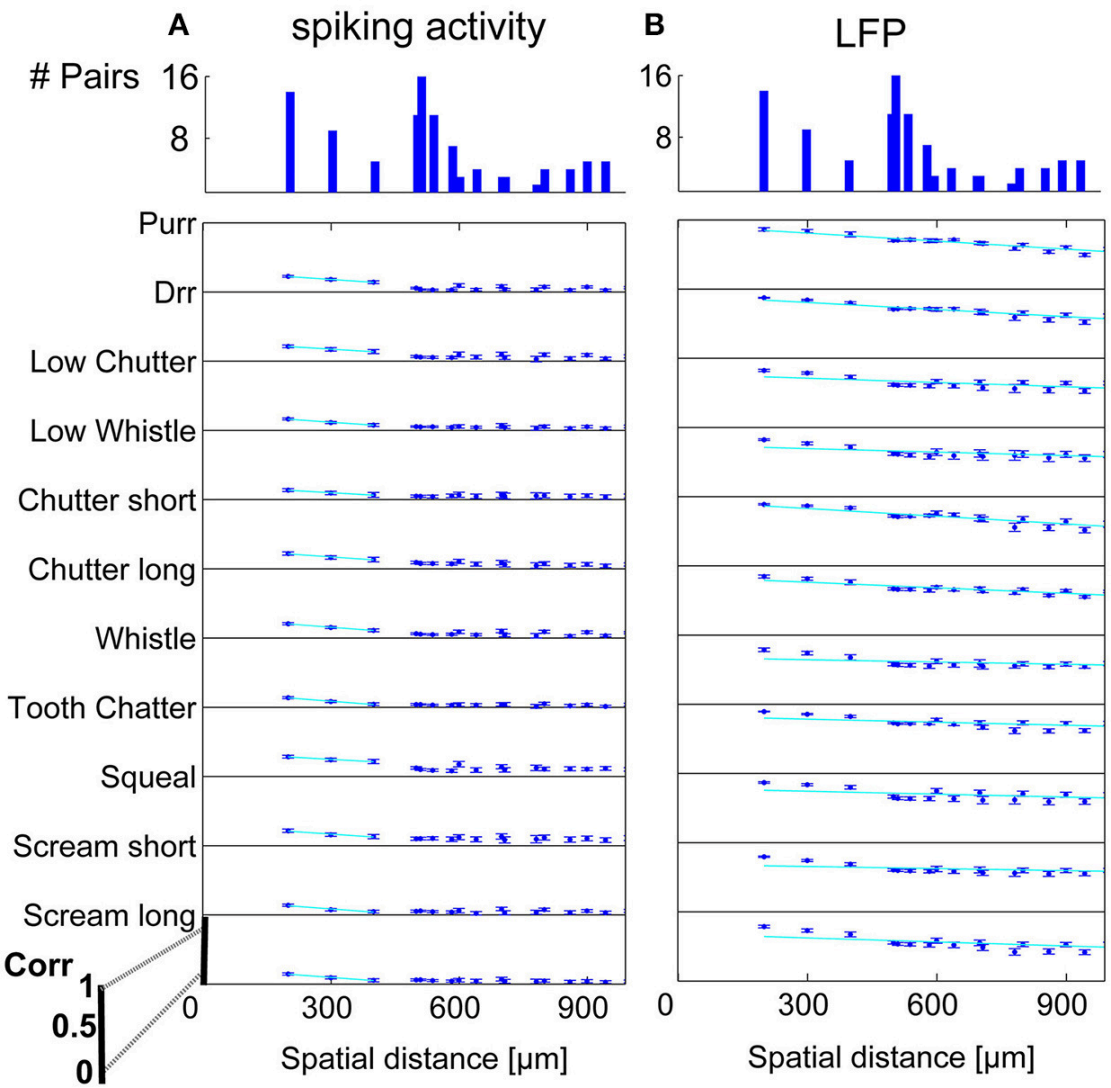
**Correlation dependence on spatial distance for similarly frequency-tuned neurons**. Averaged correlations values for each distance from one recording set are displayed for all vocalizations, for **(A)** spiking activity, and **(B)** LFPs. Exponential regression of this decrease (–), with overlaps of 41–100% see Table 1. On top, the number of multi-unit pairs for each spatial distance over which the average correlation was computed is displayed. Distances of 200–1000 μm for similarly tuned neurons are analyzed. The y-axis for each vocalization shows a correlation range of 0–1.

In order to display the relation between correlation values and the spatial distance between neurons, the values were averaged for multi-unit clusters for each spatial distance of one recording set. The relations are shown for spiking activity and LFPs, an example for one recording set of pairs with different (Figure 6) and similar frequency tuning (Figure 7) is given. Correlations decrease with spatial distance and are almost zero for distances above 400 μm for the spiking responses. LFP correlations are overall higher than those for spiking activity and display a less rapid decrease with distance, correlation values of about 0.5 still exist for the maximum measured distance of 1600 μm (Figure 6). LFP is long range activity and correlations are present over large distances. For multi-unit clusters from one recording set that have similar frequency tuning this decrease is also observed (Figure 7). These findings are consistent across all 36 analyzed recording sets. Neural and stimulus correlations follow the same decrease as the response correlations. The decrease can be approximated with an exponential function *f*(*x*) = *a*·*e*^−*bx*^, with *x* as the spatial distance. Table 1 displays the values for *a, b* and the match of data and fit χ.

**Table 1:** **Parameter for exponential regression**.

**Activity**	**Frequency tuning**	**a**	**b[1/μm]**	**χ[%]**	**〈χ〉[%]**
Spike	Different	0.01–0.59	0.57–0.0069	84–100	91
LFP	Different	0.76–1	0.012–0.003	61–94	80
Spike	Similar	0.01–0.39	0.48–0.0041	95–100	98
LFP	Similar	0.73–1	0.042–0.002	42–93	67

f(x) = a·e^−bx^, with spatial distance x, for Figures 6A,B, 7A,B.

**Figure 8:**
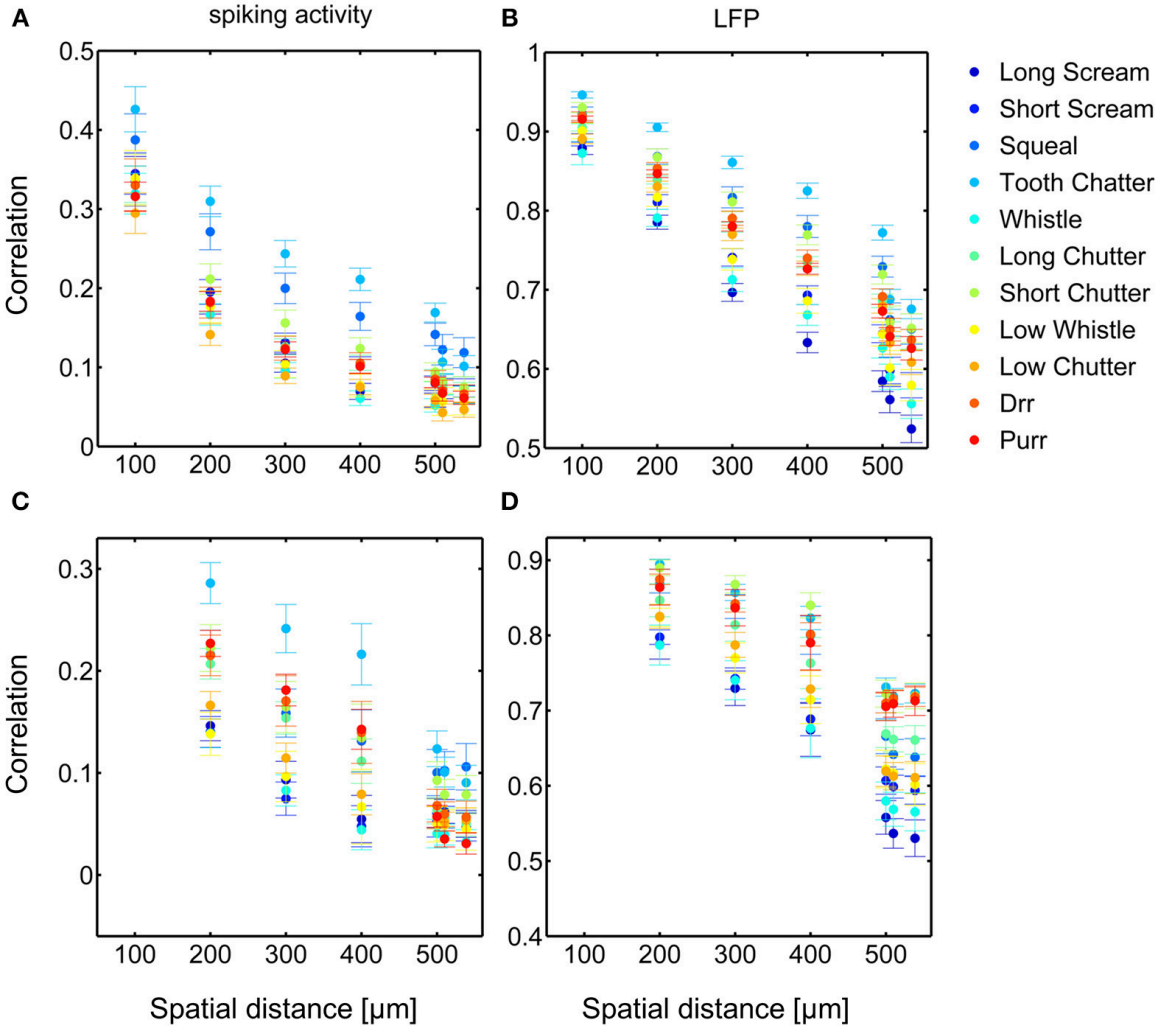
**Comparison of correlations for all vocalizations**. Correlations are shown for differently **(A,B)** and similarly **(C,D)** frequency-tuned neurons for spiking activity **(A,C)** and LFPs **(B,D)**, displayed in Figures 6, 7. Differences in total value of response correlations exist across vocalizations. For each vocalization all correlations display an almost linear decrease with spatial distance between neurons.

Dependencies on the spatial distance differ across vocalizations. Figure 8 shows the correlations displayed in Figures 6, 7 in a smaller window for distances up to 650 μm for all eleven vocalizations for spiking and LFP activity. The “tooth chatter” shows overall highest response correlations. A clear correlation decrease is observed from 200 to 500 μm for differently (Figures 8A,C) and similarly (Figures 8B,D) frequency-tuned neurons for all vocalizations.

Similarity of responses decreases almost linearly with spatial distance, and is almost zero for distances above 400 μm for spiking activity, whereas LFPs show stronger and longer range correlations even for the maximum measured distance. The parameters for the decrease are within the same range for similarly and differently frequency-tuned neurons, but the decrease is smaller for LFP than for spiking activity (Table 1). This gradual decrease within similar and different frequency regions suggests that the neural response is strongly influenced by the gradual organization of spectral and temporal preferences in the ICC.

### 3.4. Neural correlations

Comparison of correlations from simultaneous responses (response correlations) to those of non-simultaneous responses (stimulus correlations) gives an indication of the amount of neural correlations due to common input and synaptic connections leading to interactions between the multi-unit clusters. It has been shown previously that the sum of the stimulus and neural correlations does not necessarily yield the response correlations (Melssen and Epping, 1987).

**Figure 9:**
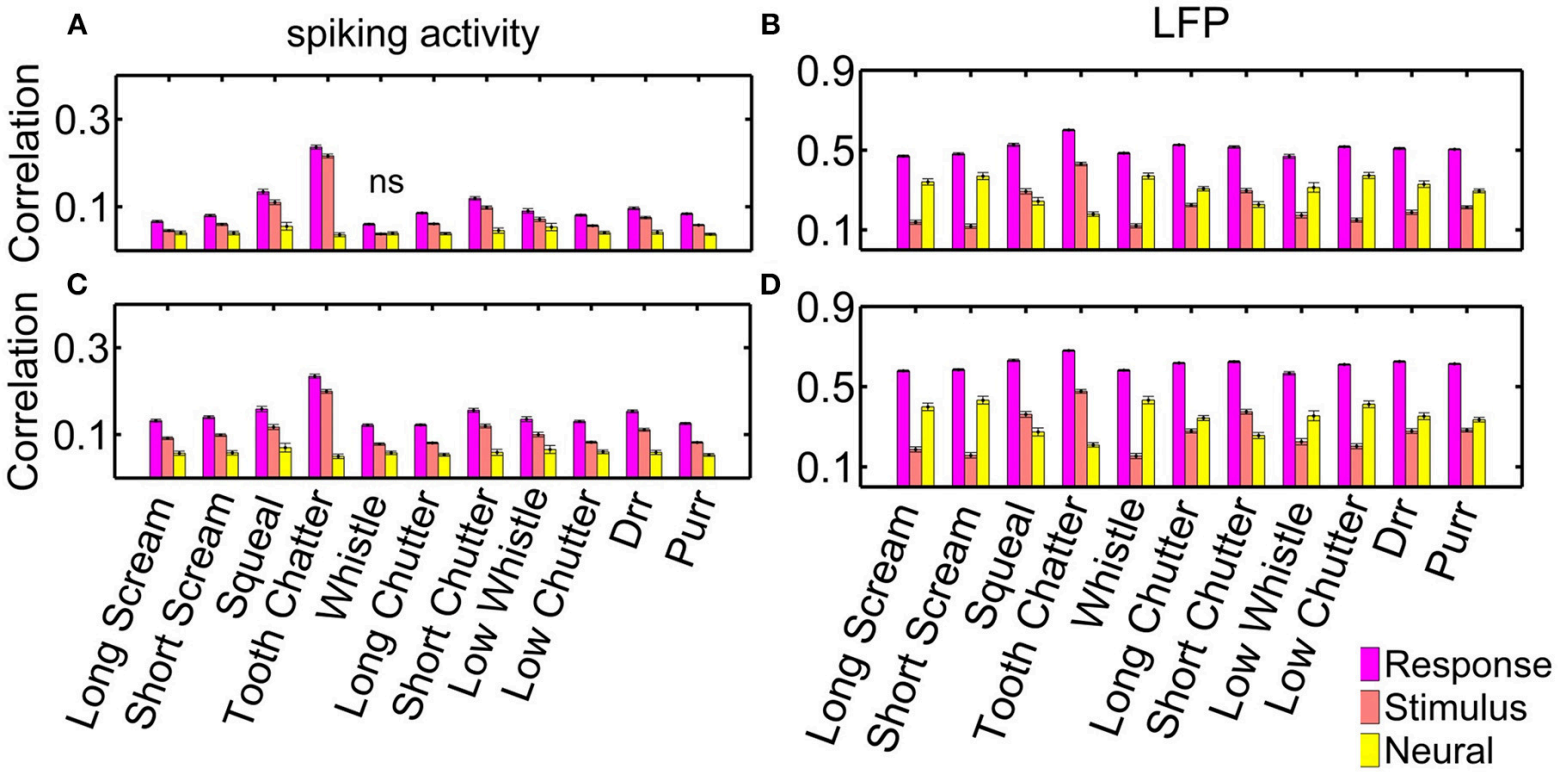
**Response, stimulus-driven and neural correlations**. Correlations for spiking activity, for **(A)** differently (*n* = 10,775 pairs); and **(C)** similarly frequency-tuned neurons (*n* = 4230 pairs); and correlations for LFP activity for **(B)** differently, and **(D)** similarly frequency-tuned neurons. Non-significant differences are denoted by ns, all other differences are significant (*p* = 0.05).

Figure 9 displays all three types: the averaged response correlations, the correlations due to the stimulus and the neural correlations, for multi-unit pairs with either similar or different frequency tuning, both, for spiking (a) and LFP (b) activity. The displayed error was obtained via error propagation. Correlation values vary across multi-unit clusters as displayed in Figure 5.

Response correlations of the spiking activity are significantly larger than non-simultaneous correlations, and these are larger than the neural correlations. The difference between response, stimulus and neural correlations is significant for all vocalizations for spiking and LFP activity (Figure 9). Only the “whistle,” which elicits the overall lowest response correlations, does not show significant differences between the stimulus and neural correlations of spiking activity for differently frequency-tuned multi-unit cluster pairs, Figure 9A. Differences between response and stimulus correlations are not significant for all vocalizations within each recording set, but are significant when averaged across all multi-unit clusters.

Differences between response, stimulus and neural correlations are larger for LFP than for spiking activity. These are significant for correlations of all vocalizations in single recording sets and for averaged values across all multi-unit clusters. For the LFP activity, neural correlations are even significantly higher than stimulus correlations for all vocalizations but three, for both, similarly and differently frequency-tuned multi-unit pairs (Figure 9B). The three exceptions, the “tooth chatter,” “short chutter,” and “squeal,” show energy across the whole frequency range at short time intervals in the vocalization spectrograms, see Figure 3 and Lyzwa et al. (2016). For the spiking activity, these three vocalizations also show the overall smallest amount of neural correlation relative to the response correlations.

To summarize, neural correlations which are most likely due to common input and synaptic connections leading to interactions between multi-unit clusters exist in the central inferior colliculus for spiking activity and for local field potentials. These correlations exist between similarly and differently frequency-tuned multi-unit activity. They are significant but minor for spiking activity, and much larger for LFPs which are long range activity (Pettersen et al., 2012). Neural correlations exist in the inferior colliculus. Thus, the responses are also shaped by the interactions between neurons.

**Figure 10:**
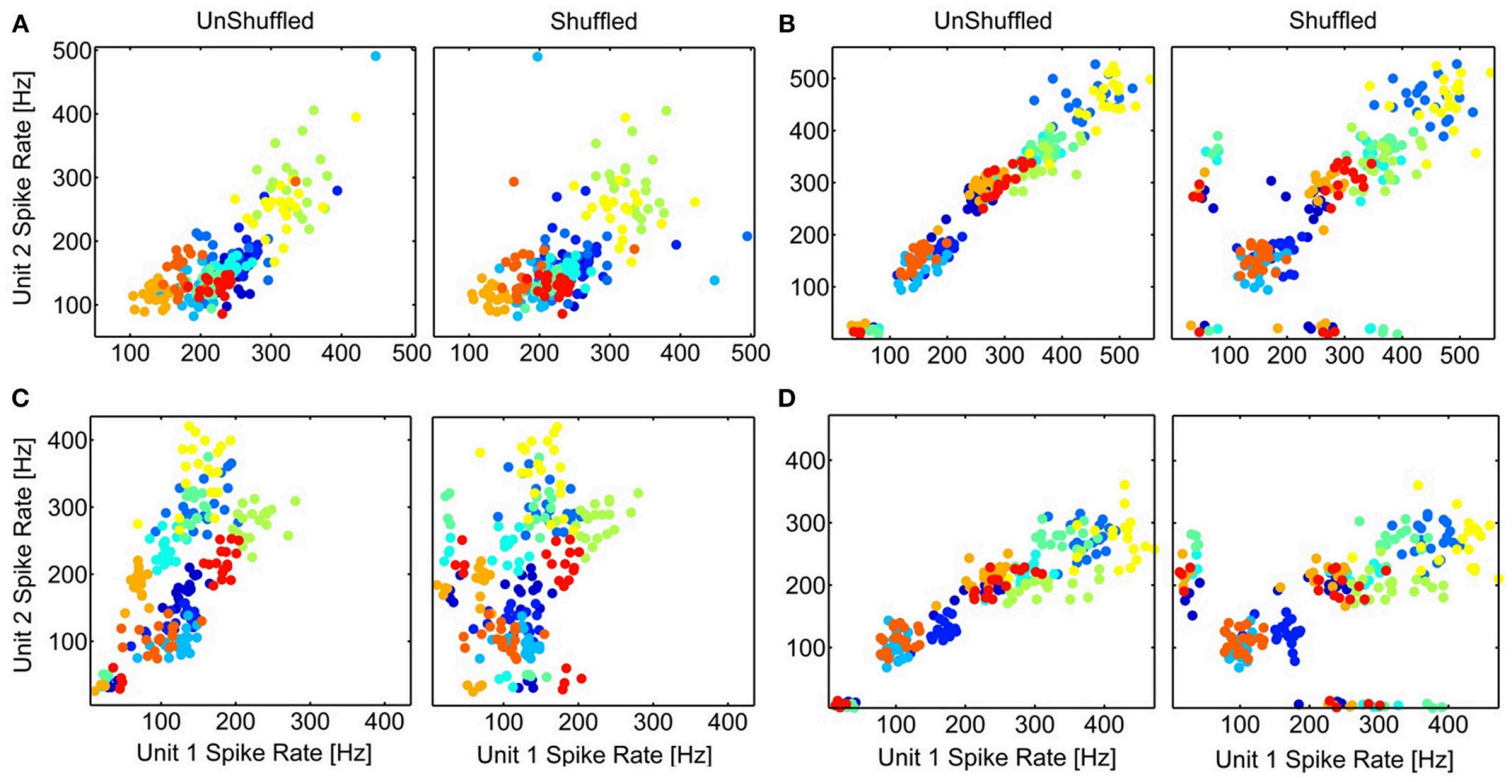
**Separation of spike rates with correlated trial-variability**. The scatter plots of the average spike rates in response to the 11 vocalizations for simultaneous (UnShuffled) and non-simultaneous (Shuffled) trials are compared for 4 multi-unit pairs, for units with different **(A,C)** or similar frequency tuning **(B,D)**; and which are spatially relatively close **(A,B)** or far away **(C,D)**. **(A)** *BF*_1, 2_ = 5 *kHz*, 8 *kHz*; 360 μ*m*; **(B)** *BF*_1, 2_ = 2.25 *kHz*, 2.83 *kHz*; 400 μm; **(C)** *BF*_1, 2_ = 8 *kHz*, 2.25 *kHz*; 1270 μ*m*; **(D)** *BF*_1, 2_ = 2 *kHz*, 2.25 *kHz*; 800 μ*m*. The colors for the different vocalizations correspond to those in Figure 8.

Interactions between neurons can lead to a co-variation of their trial-to-trial variability of the temporal spiking responses, which might also manifest in a correlated trial-to-trial variability of their spike rates. The averaged stimulus-elicited spike rate for each trial (*n* = 20), for multi-unit cluster pairs is compared for simultaneous (response correlations) and non-simultaneous responses (stimulus correlations) to investigate whether a decorrelation (shuffling) induces better separability of the spike rates to different vocalizations. Thus, we explored whether the contribution of neural correlations to separate spike rates of different vocalizations differed among multi-unit cluster pairs with a small or large difference either in frequency tuning, or in spatial distance. Figure 10 displays examples of scatter plots for multi-unit pairs with similar and different frequency tuning, and for distant and relatively close-by multi-unit clusters. Although shuffling changes the distribution of responses, separability of the 11 vocalizations does not change substantially in any of the cases. This is true across all recording sets and was shown here for 4 examples. Correlated trial-to-trial variability due to interactions between neurons in the ICC does exist but does not alter separability of the multi-units' spike rates to vocalizations, although at a single neuron level this might be different.

## 4. Discussion

In this work, we analyzed similarity of multi-unit responses to a set of 11 vocalizations across the mammalian central inferior colliculus. Our findings are based on a large set of multi-unit clusters (*N* = 1152), of which the best frequencies span a range between 0.5 and 45 kHz. The studied vocalizations are a representative set of behaviorally relevant stimuli (Berryman, 1976).

Neurons with similar frequency tuning (1/3 octave) have higher correlation values than differently tuned neurons (Figure 5) but differences were minor and correlation varied across multi-unit clusters; hence, the frequency selectivity made almost no contribution to the correlations between pairs of units. We investigated response similarity separately for these two groups.

### 4.1. Spatial dependence

This work is a first systematic investigation of the dependence of response similarity on spatial distance in the mammalian ICC (Epping and Eggermont, 1987; Eggermont, 2006; Chen et al., 2012).

Response correlations and neural correlations to artificial sounds have been analyzed for single neurons in the cat ICC, for distances up to 370 μm (Chen et al., 2012), showing that nearby neurons have a higher probability of displaying similar neural preferences and responses, and suggesting a microcircuitry. Epping and Eggermont (1987) analyzed neural activity of 150 sorted units in the auditory midbrain of the grass frog. Using cross-correlation, they found response correlations for 60% of the neurons and 15% of the neurons displayed correlations due to neural interactions and connections. These neural correlations were restricted to pairs with distances less than 300 μm, whereas the response correlations were independent of the spatial distance. The authors suggested that the high amount of response correlations relative to the small amount of neural correlations indicates that in order to create the neural response, the stimulus input plays a predominant role over neural mechanisms (Epping and Eggermont, 1987). They attributed this to a spread projection (rather than to restricted areas) of the stimulus input which would be in line with a weak tonotopic organization in the grass frog midbrain and to the finding that neighboring neurons often display different spectral preferences.

In contrast to the grass frog midbrain, the mammalian ICC has a clear tonotopic structure with neighboring neurons displaying similar spectral preferences. The correlation structure as described by Epping and Eggermont is very different for the mammalian ICC. We showed that response similarity depends on the spatial distance between two multi-unit clusters, and decreases exponentially with increasing distance. In general, for distances above 400 μm (Figures 6A, 7A, 8A,C), very little (≤ 0.11) correlation of spiking responses is present. Correlations vary across vocalizations but all display the linear dependence on spatial distance between neurons (Figure 8). The “tooth chatter” which shows phase-locking to the stimulus envelope throughout a very large frequency range (Figure 4), displays the highest correlations. Responses from local field potentials have overall higher correlations, and a less steep decrease and display high correlations (>0.5) for distances as high as 1600 μm (Figures 6B, 7B, 8B,D). These large correlation lengths are due to the local field potential being long range activity.

The decrease in this example of a recording could be exponentially fitted with amplitude and decay parameters ranging between *a* = 0.01−0.59 and *b* = 0.041−0.57/μm for spiking activity, and the ranges for similarly and differently frequency-tuned neurons overlapped in parts (Table 1). In a higher auditory processing station, in the cortex, a correlation dependence on the spatial distance has also been demonstrated. Eggermont (2006) analyzed neural groups, that reflect patched activity and were termed “clusters,” in the cat primary auditory cortex with the use of cross-correlation matrices of spontaneous activity. The author found that the correlation followed an exponential decrease with distance, *a* = 0.05, *b* = 0.24/mm. Our obtained values for the maximum amplitude fall within the same order, but the decay parameter *b* obtained in our study is at least 17 times larger than the one found by Eggermont (2006). Eggermont found this dependence for spontaneous activity of neural groups in the cat primary auditory cortex which is also tonotopically organized. The steeper decrease in our work might be explained by the smaller size and mapping space available for the ICC compared to the primary auditory cortex, and by the different and smaller animal studied. It has been suggested that the functional organization is dynamic, and that the functional connections depend on the particular stimulus applied (Epping and Eggermont, 1987). Thus, the use of responses to natural communications sounds instead of responses to spontaneous activity could also account for this difference in spatial decrease.

In this analysis, correlations for pairs of two multi-unit clusters with similar frequency tuning within 1/3 octave that are separated by at least 200 μm were analyzed (respectively 100 μm for differently tuned neurons), in order to ensure comparison of different signals. However, neural responses from a radius of more than 200 μm might be picked up by the recording electrode. In order to exclude this possibility, and to investigate correlation dependencies for smaller distances (<100 μm), single neuron recordings could be used. In the present analysis, we compare within 1/3 octave similarly and differently frequency-tuned multi-unit cluster. This allows one to make inferences about the organization of response similarity within the ICC insofar as neurons within an isofrequency laminae have the same best frequency within this interval, hence if the neurons differ in best frequency by more than 1/3 octave, they are likely located in different isofrequency laminae. However, histological stains would allow one to obtain the exact location of the multi-unit clusters within the ICC. Furthermore, knowing the positions of the neurons would allow one to map out and test the dependence of the response similarity on the specific location within the ICC. Additionally, this study could be complemented by single neuron responses which would further reduce ambiguity of the exact position of the neural response. Another interesting and challenging investigation would be to label the synaptic input to the ICC from the different ascending brainstem nuclei and measure the position of the neuronal pairs relative to these main ICC inputs.

We analyzed response similarity for multi-unit clusters with either similar or different frequency-tuning and showed a minor but significantly higher correlation for similarly frequency-tuned neurons (Figure 5) but with variability. It would be informative to study the similarity dependence on spatial distance with respect to the best amplitude modulation frequency of the neurons. This might help to further discriminate in detail response similarity within and across isofrequency laminae. An ideal experiment to measure this would be to record simultaneously from several neurons within an isofrequency lamina using tetrode electrode arrays with relatively high impedances to capture single neuron activity. Recordings would be made in response to vocalizations and in response to dynamic moving ripple sound (Escabí and Schreiner, 2002) which allows one to compute the receptive fields and modulation preferences for the single neurons. Histological stains would inform one about the actual positions of the recording sites.

In summary, despite the vocalizations displaying very diverse and inhomogeneous spectral contents, and despite the locally diverse inputs to the ICC, the neural representations of vocalizations exhibit gradual organization of the similarity of the neurons' responses. Multi-unit clusters with similar spiking responses are spatially spread for distances <400 μm, however, differences might exist for single neurons and cannot be captured at this resolution level. These findings give indications for applications in auditory midbrain prosthesis, e.g., for the sufficient spatial separation between stimulating electrodes and contacts.

### 4.2. Neural correlations

For our large set of 1152 multi-unit clusters, we find that response correlations are significantly higher than the respective stimulus correlations. Neural correlations due to common input and synaptic connections leading to neural interactions exist in the mammalian ICC. This finding is in contrast to a study of Epping and Eggermont (1987) in which they showed that for the grass frog midbrain only 15% of the units displayed neural correlations. In contrast to the grass frog midbrain, it has been shown that the mammalian ICC has a clear tonotopic structure with neighboring neurons displaying similar spectral preferences, and a gradient of amplitude modulation frequency. We showed significant neural correlations across the ICC in response to species-specific sounds. The correlations also show a linear decrease with spatial dependence. Thus, in the mammalian ICC neural interactions contribute significantly to shape the organization of neural responses to vocalizations and the output of this nucleus. Epping and Eggermont (1987) also found stimulus dependencies for half of the neural correlations, indicating that the functional organization is dynamic, and that the functional connections depend on the particular stimulus applied. In this work, we analyzed correlations separately for each vocalization, in order to account for such possible stimulus dependencies. The amount of neural correlations varies across vocalization stimuli (Figure 9), which is also true for the response correlations (Figure 5).

The neural correlations, although smaller than the response correlations, follow the same spatial dependence, which is also true for the stimulus correlations. This could indicate that both effects contribute to the gradual decrease of response similarity, the gradual changing of spectrotemporal properties with spatial distance as well as neural interactions which decrease with spatial distance, since the interactions are most likely between nearby and synaptically connected neurons. The neural correlations do not contribute to a better separability between the time-averaged spike rates across similarly and differently frequency-tuned, distant or close-by neural pairs (Figure 10), but might further shape the organization of the neural representation of the sensory stimulus within the ICC.

Single neuron responses could be additionally recorded for this study of large spatial distances. Single neuron recordings would allow one to make inferences of connectivities and quantify the amount of correlations due to neural interactions. However, limitations of the shift predictor exist even for single neurons such as the obscuring of neural correlations due to deterministic responses or temporal overlap of stimulus and neural response (Epping and Eggermont, 1987). Probabilistic models based on pairwise interaction, describing the weights of interactions in a network could be established (Schneidman et al., 2006).

Furthermore, anesthesia has been shown to affect neural responses (Astl et al., 1996), and fluctuating or slowly changing anesthesia levels also change the brain state which might affect the cross-correlation strength. Thus, response similarity and particularly neural correlations might differ in awake animals, although recordings in awake animals also bear difficulties and biases. Since anesthesia has non-negligible effects on the neural activity, correlation values are likely to improve in awake animals.

In summary, it was found that multi-unit clusters in the ICC display significant neural correlations due to common input and synaptic connections leading to interactions of the neurons. These exist for similarly and differently frequency-tuned neurons, decrease with spatial distance and differ across vocalizations. For LFPs, the neural correlations are even larger than stimulus correlations for most of the vocalizations. These findings suggest that the neural interactions shape the spiking output of this nucleus, and the neural representation of vocalizations with gradually decreasing similarity in the ICC.

In conclusion, we showed that, despite the diverse inputs to the ICC from all ascending projections, terminating in different spatial locations in the ICC (Oliver, 2005), and the rich spectrotemporal properties of vocalizations, the neural representation shows an organization of gradual decrease in response similarity with spatial distance, for spiking activity and for local field potentials. Thus, when comparing responses of neuronal groups to complex sound, such as vocalizations across the ICC, it is important to take their spatial separation into account and not only their frequency tuning. Our findings suggest that, for multi-unit clusters in the mammalian inferior colliculus, the gradual response similarity with spatial distance to natural complex sounds is shaped by neural interactions and the gradients of neural preferences.

## Author note

This paper is based on a chapter in the Ph.D. thesis of DL which had been published under the Common License agreement. https://ediss.uni-goettingen.de/handle/11858/00-1735-0000-0022-6026-D.

## Author contributions

DL conceived the work, performed data analysis and wrote the manuscript. DL and FW interpreted the data and revised it critically for important intellectual content.

### Conflict of interest statement

The authors declare that the research was conducted in the absence of any commercial or financial relationships that could be construed as a potential conflict of interest.
